# Flipped Classroom With Artificial Intelligence: Educational Effectiveness of Combining Voice-Over Presentations and AI

**DOI:** 10.7759/cureus.48354

**Published:** 2023-11-06

**Authors:** Marcos Sanchez-Gonzalez, Mark Terrell

**Affiliations:** 1 Health Services Administration, Lake Erie College of Osteopathic Medicine, Erie, USA; 2 Medical Education, Lake Erie College of Osteopathic Medicine, Erie, USA

**Keywords:** medical education curriculum, online medical education, customized medical education, ai in medical education, higher education medical training

## Abstract

Background: Most theorists and medical educators agree that a curriculum rich in active learning (AL) strategies, such as a flipped classroom, is superior to passive listening to promote better retention and application of new knowledge. Although AL multimodal teaching strategies have been considered the most effective, including online virtual teaching, voice-over pre-recorded lectures, and, more recently, the addition of artificial intelligence (AI), data on the effectiveness of these methods in medical education is scarce. The present educational research study examined the effectiveness of voice-over-style lectures and AI in facilitating learning outcomes as assessed by test scores after participating in basic science lectures in a medical school setting.

Methods: Participating students were divided equally into two educational strategy groups: slide decks only traditional way (PPT) or PPT plus AI (PPT+AI) platform (edYOU; Los Angeles, CA, USA). The PPT+AI group comprised the PPT with narration and real-time interaction with an AI being personalized, which leverages natural language processing to tailor customized conversations to each student’s current knowledge. Students in the two groups were asked to participate in a formative quiz (not reflective of their academic evaluations) to answer questions relevant to voice-over lectures (PPT and PPT+AI). The statistical strategy for conducting quiz item analysis included item difficulty, item discrimination, and point-biserial correlation R. A student's T-test was conducted to compare the two strategies’ effectiveness via test scores. A priori, an alpha level of 0.05 was considered significant.

Results:* *Data are presented as mean ± s.e.m.; Cohen’s *d*. A total of 42 (n=21 in each group) students participated in the study. Students using PPT+AI obtained statistically significant (*P* <0.043; *d = .54)* higher quiz scores under challenging questions and less time spent in lectures (54.1 ± 14.3 hrs.) in the PPT+AI group (P <0.001;* d* = 1.17) compared with the PPT group.

Conclusions: The PPT+AI strategy could be the difference between a pass and a fail, as the PPT+AI strategy is particularly efficient in improving difficult question test scores. At the same time, students may learn the material in less time (efficiency). Research on the application of AI as part of educational strategies for improving satirized test scores, including boards, is warranted. The present study is part of the necessary early steps to better understand the impact of AI as an educational strategy for improving educational outcomes.

## Introduction

Although multimodal teaching strategies have been considered the most effective, most theorists and medical educators agree that a curriculum rich in active learning (AL) strategies is superior to passively listening to the information in lectures in promoting better retention and application of new knowledge [1]. AL comprises various learning activities such as flipped classroom, think-pair-share, turn and talk, and bulleted breaks during lectures requiring learners to construct, understand, and comprehend the knowledge derived from their educational experience “while simultaneously improving knowledge gain and recall abilities” [1,2]. First introduced in the late 90s, the flipped classroom educational strategy has been accentuated since, owing to its proven effectiveness in educational settings. Characterized by using allocated didactic time for active learning, the flipped classroom is ideal for focusing on developing the application of material, a better understanding of concepts, and improving standardized testing scores. For this reason, it has been recently recognized that adopting a multimodal approach when selecting instructional methods is the best strategy to increase the chances of success of the educational activity [3,4]. Hence, educational research suggests that the best way to teach modern is by combining multiple pedagogical resources to complement one another, where students learn more effectively when multimodal and system-based approaches are integrated or supplement each other [5].

Despite being developed in 1955, the use of artificial intelligence (AI) applications has increased exponentially over the past few years, a revolution that is posited to add tools to enhance medical education [6]. Educational research suggests that AI’s primary uses in medical education are learning support, assessment of students’ learning, and, to a minimal degree, curriculum review [6]. Providing constant (24/7) feedback and a guided learning pathway might be possible by adding AI technologies as part of the educational tools. Subgroup analysis revealed that medical undergraduates are one of the primary target audiences for AI use. However, educational research to assess the usefulness of this technology is lacking. Accordingly, the goal of the present educational research study was to test the effectiveness of both voice-over style lectures and AI technology to facilitate learning. It was hypothesized that adding AI to the flipped classroom would improve academic performance.

## Materials and methods

Study population

The subject population was incoming medical students from California North State University, College of Medicine (CNUCOM), Class of 2027 (first-year students). The inclusion criteria were first-year students enrolled in the Faculty of Medicine without previous knowledge of basic sciences, including cell biology, immunology, biochemistry, pharmacology, microbiology, histology, pathology, and medical terminology. The learning methodology was to have preloaded lectures available for students to watch independently and use the allotted lecture time for active learning. The Institutional Review Committee for Research approved the study, allowing students to be recruited from the CNUCOM via email through the Office of Admissions. 

Design

Participating students were randomly assigned into two educational strategy groups: slide decks only the traditional way (PPT) or PPT plus AI (PPT+AI) platforms (edYOU; Los Angeles, CA, USA). Randomization and matching were performed by someone not associated with assessing the students using a computer-generated random number table (with a 20% random element) using an allocation ratio of 1:1, as previously described [7]. The PPT+AI group comprised the PPT with narration and real-time interaction with an AI being. Students in the two groups were asked to participate in a formative quiz (a 15-item multiple-choice instrument not reflective of their academic evaluations) and answer usability-related questions relevant to voice-over lectures (PPT and PPT+AI). In addition, surveys were administered electronically to gauge feedback and general satisfaction or dissatisfaction with the PPT and PPT+AI sessions. The survey included questions to account for confounding variables, such as attention paid while using voice-overs or outside resources for the content in question. The surveys were administered after they had experienced PPT or PPT+AI. In the present study, the learning outcomes were divided into theoretical (quiz scores) and satisfaction contexts (usability of the learning platform).

Artificial intelligence platform

The AI learning experience was delivered by proprietary technologies designed to be personal, ethical, and educationally effective (edYOU; Los Angeles, CA, USA). using a personalized ingestion engine (PIE), the platform curates diverse learning materials from expert sources worldwide. A personalized AI (PAI) then leverages natural language processing to tailor customized conversations to each student’s current knowledge. In addition, an intelligent curation engine ensures that the AI interacts safely using techniques like content flagging, toxicity blocking, and data verification. This combination enables AI tutors on the platform to build long-term mentoring relationships by adapting to each learner’s evolving needs.

Quiz item analysis

The statistical strategy for conducting item analysis included item difficulty, item discrimination (ID), and point-biserial correlation R (PBI) [7]. Briefly, the item difficulty was calculated by looking into the proportion of the total learners answering correctly; this metric’s target was 50%-70% (valid difficult questions). The item discrimination accounts for the difference between the upper quartile (of total scores) who answered correctly vs. the lower quartile of those also answering the item correctly; the main goal was to avoid negative scores (invalid questions). Lastly, the PBI was performed via the correlation between test and item scores; the desired target was over 0.20. Items not meeting the criteria described above were considered non-valid and hence nullified.

Statistical analysis

Student’s T-test and Cohen’s d calculation ([M2 - M1]⁄SDpooled) were performed to compare the effectiveness of the different educational strategies (PPT vs. PPT+AI) and estimate the effect size, respectively. A priori, an alpha level of 0.05 was considered significant. The interpretation of effect sizes as small (d = 0.2), medium (d = 0.5), and large (d = 0.8) was based on benchmarks suggested by Cohen [8].

## Results

Usability surveys

All of the students in the PPT+AI group were naive about using an educational platform for AI. Most of the students in the PPT+AI group found that the application was helpful, with interactions generally accurate in the context of their questions (~60%). User satisfaction and usability were 4.11 and 4.33 on a scale from 1 (low) to 7 (high), respectively.

Quiz scores

**Figure 1:**
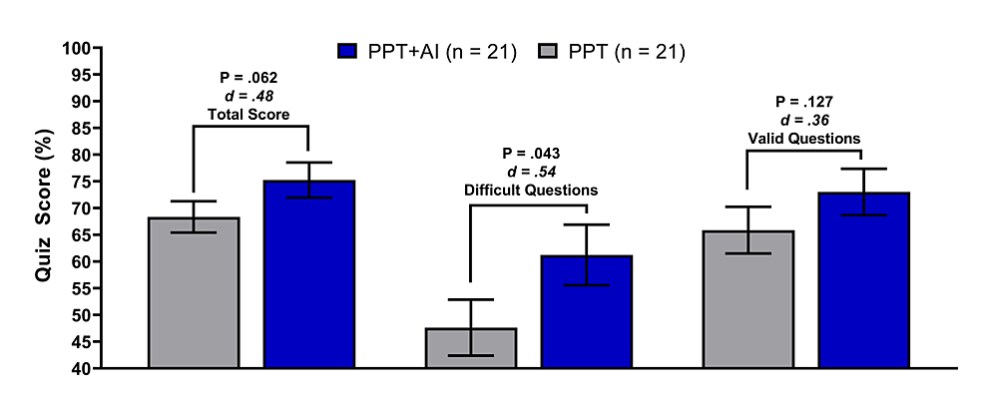
Test scores comparison between the two educational strategies. Note: PPT, slide decks only, is the traditional way; AI, artificial intelligence platform.

**Figure 2:**
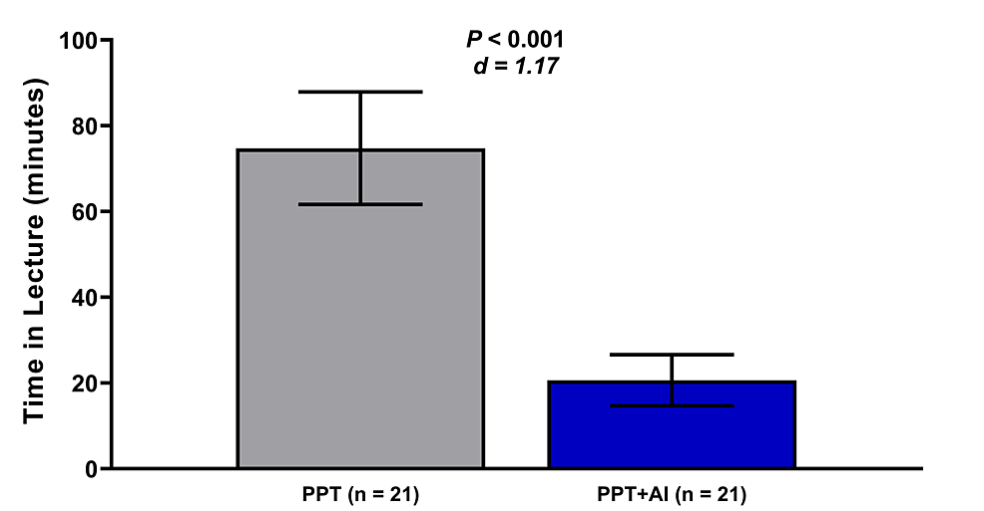
Time spent in lectures. Note:  PPT, slide decks only, is the traditional way; AI, artificial intelligence platform.

Data are presented as mean ± s.e.m.; Cohen’s d. A total of 42 (n=21 in each group) students aged 18-28 participated in the study. Students using PPT+AI obtained statistically significant (P <0.043; d = .54) higher quiz scores (13.6 ± 7.7%; Figure 1) under challenging questions and statistically significant (P <0.001; d = 1.17) less time spent in lectures (54.1 ± 14.3 min; Figure 2) in the PPT+AI group compared with the PPT group. While the students in the PPT+AI, on average, scored a passing grade on the quiz (75.2 ± 3.3%), there were no statistical differences (P <0.062; d = .48) compared with the PPT (68.3 ± 2.9%) (Figure 1). A similar trend in score differences was observed when only the valid questions per item analysis were considered, such that the PPT+AI score was not statistically (P <0.127; d = .36) higher (7.1 ± 6.2%).

## Discussion

The present educational research examined the effectiveness of both voice-over-style lectures using AI technology to facilitate learning in basic science courses. The PPT+AI was superior to the traditional PPT for improving academic performance in medical students undertaking basic sciences courses. AI applications may provide the means to promote a more engaging teaching and educational environment conducive to improving academic performance.

Research suggests that AI’s primary uses in medical education are learning support, assessment of students’ learning, and, to a minimal degree, curriculum review [6]. The use of artificial intelligence (AI) in medical education has the potential to facilitate what would be complicated tasks and improve overall teaching efficiency [9]. For example, AI could help automate written response assessment, or provide reliable feedback on medical management plans and imaging, provide constant (24/7) feedback, and provide an improved guided learning pathway, among other applications. In addition, AI education-driven platforms may prepare learners one-on-one in a simulated classroom or clinical setting to study and prepare them for tests, clinical encounters, and specific clinical skills.

The effectiveness of AI as part of educational strategies to improve academic performance has been recently highlighted in a systematic review conducted by Varma et al. (2023) [10]. While AI applications in medical education are categorized into teaching, assessing, and trend spotting, the review demonstrated that AI is a feasible supplement to undergraduate medical curricula. Interestingly, studies directly comparing AI to current teaching methods have documented favorable results [11,12]. Moreover, AI-enhanced lectures have increased positive feedback between 18%-21% in pre-clinical microbiology [11]. Similarly, a computer tutor computer program (CIRCSIM-Tutor) designed to conduct a natural language dialogue with a medical student demonstrated that significant learning occurs during a 1-hour interaction with the program [12]. In their study, Michael et al. used a pretest/post-test assessment strategy to test for program educational efficacy, while students indicated considerable satisfaction with the CIRCSIM-Tutor [12]. If taken together, the results of the prior studies suggest that interactive strategies, including AI, can seamlessly be added to the teaching and lecture delivery to improve academic performance and satisfaction, which are in line with the present study’s main observations. 

As with any study, the present educational research work is not exempt from limitations. First, a limited sample size affected the statistical power as total test scores and valid question scores were not statistically different despite moderate effect sizes (d = .36-.48). It is worth mentioning that the students in the PPT+AI scored passing grades. Another limitation is that the study did not use a pretest-posttest paradigm that could be a superior design to demonstrate educational strategy efficacy. Additionally, the lectures were limited to basic science and not clinical content, limiting the generalization. Lastly, in this study (proof-of-concept), the machine learning capabilities of the AI program were not ultimately used as it was the first cohort of studies using the system. Hence, part of the prospective studies and cohorts of students would benefit from faster reaction times from the AI, better interactions, and more direct answers to users’ doubts or questions about the main concepts studied.

## Conclusions

In sum, multimodal educational strategies involving AL with the addition of AI were superior to the traditional PPT for improving academic performance in medical students. Many teachings methodological improvements could be obtained by AI adoption, as it is in the medical profession and medical education. AI applications may provide the means to promote a more engaging teaching and educational environment as part of AL strategies. Educational research aimed at introducing AI into the medical school curriculum for medical professionals to better understand AI algorithms and maximize their use in medical education is warranted. 
